# Rewiring transcriptional plasticity: the case for dual YAP1 and TAZ targeting in gastric cancer peritoneal metastases

**DOI:** 10.47248/chp2603010004

**Published:** 2026-03-28

**Authors:** Rahul Kumar

**Affiliations:** Department of Pharmacology and Therapeutics, Roswell Park Comprehensive Cancer Center, Buffalo, NY 14263, USA

As key transcriptional co-activators, YAP1 and TAZ are frequent drivers of progression across a broad spectrum of solid tumors. These mechanosensitive regulators respond to tumor microenvironmental cues to fuel essential malignant traits such as proliferation, stem cell-like plasticity, drug resistance, and metastasis [1]. While normal tissues typically suppress their activity, cancers, driven by inflammation and Hippo pathway disruption, frequently exhibit sustained YAP1/TAZ signaling. Because many malignant tumors depend on this axis to maintain their aggressive phenotype, targeting YAP1 and TAZ represents a rational and high-priority therapeutic strategy [1].

In this issue of *Cancer Heterogeneity and Plasticity*, Shumei Song and colleagues explore the therapeutic potential of targeting the transcriptional co-activators YAP1 and TAZ in gastric cancer peritoneal metastases (GCPMs), an aggressive and clinically challenging form of advanced gastric cancer [2]. The investigators found that YAP1, TAZ, and their transcriptional partners TEAD1–4 are co-expressed, at markedly elevated levels, in tumor samples from patients with peritoneal metastases. Importantly, elevated expression of these genes strongly correlates with poor overall survival, underscoring their clinical relevance and suggesting that YAP1/TAZ-driven transcriptional programs contribute significantly to metastatic progression. A major mechanistic insight from the study is the identification of a compensatory relationship between YAP1 and TAZ. When YAP1 is inhibited, through either genetic or pharmacologic approaches, TAZ expression increases, effectively sustaining oncogenic signaling. This compensatory upregulation enables continued tumor growth and may help explain the limited efficacy observed in earlier clinical strategies that selectively targeted YAP1 alone. Thus, functional redundancy between YAP1 and TAZ represents a key obstacle to single-agent therapeutic strategies (Figure 1).

The study further demonstrates that, upon YAP1 depletion, TAZ enhances its interaction with TEAD4 and cooperates with the AP-1 transcription factor complex, specifically, the c-JUN/FOSB heterodimer, to drive transcriptional programs that promote tumor cell proliferation and invasion. These findings reveal how tumor cells unleash transcriptional plasticity by dynamically rewiring transcriptional networks to maintain malignancy in the face of targeted inhibition. Importantly, the authors show that dual inhibition of YAP1 and TAZ using antisense oligonucleotides (ASOs) is substantially more effective than targeting either gene alone (Figure 1). Combined suppression resulted in maximal inhibition of tumor cell proliferation, colony formation, and invasive capacity in vitro. These findings were further validated in vivo using patient-derived xenograft (PDX) mouse models, where simultaneous targeting of YAP1 and TAZ led to significant reductions in tumor volume and weight compared to single-agent approaches. In addition to its direct antitumor effects, YAP1 inhibition was also found to modulate the tumor immune microenvironment. Specifically, suppression of YAP1 increased PD-L1 expression, providing a rationale for combination therapy with immune checkpoint blockade. Indeed, combining YAP1 inhibition with anti-PD-1 immunotherapy produced the greatest reduction in tumor burden and enhanced infiltration of cytotoxic CD8^+^ T cells in tumor tissues, suggesting a synergistic interaction between YAP/TAZ targeting and immunotherapy.

Overall, the study concludes that co-targeting of YAP1 and TAZ overcomes compensatory resistance mechanisms and represents a promising therapeutic strategy for patients with GCPMs. This dual-targeting approach, particularly when integrated with immunotherapy, may offer improved clinical outcomes for gastric cancer patients with widely disseminated disease. The current study also raises important questions and identifies immediate future tasks. For example, what would be systemic toxicity profiles associated with the joint suppression of YAP1/TAZ, considering their critical roles in maintaining normal tissue homeostasis? As YAP1 inhibition leads to increased PD-L1 expression in gastric cancer cells, does YAP1 signaling transcriptionally represses PD-L1? Ultimately, the feasibility and potential benefits of YAP1/TAZ co-inhibition combined with checkpoint blockade in patients with GCPMs will have to be tested out in prospective clinical trials.

## Figures and Tables

**Figure 1.**
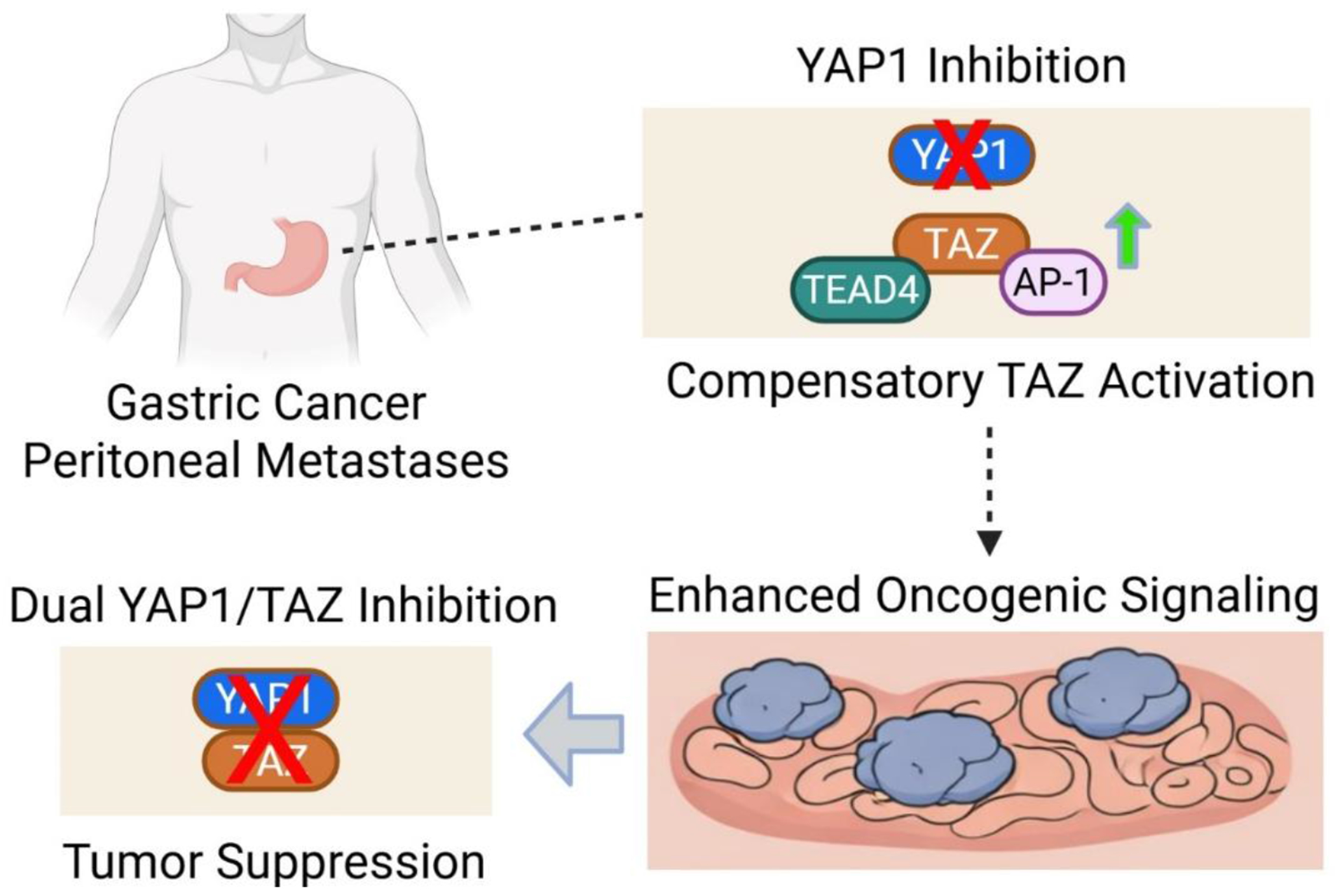
Compensatory TAZ activation following YAP1 inhibition in gastric cancer peritoneal metastases. Schematic model illustrating the effects of YAP1 inhibition in gastric cancer peritoneal metastases (GCPMs). Targeting YAP1 alone leads to compensatory activation of TAZ, accompanied by enhanced interaction with transcriptional partners such as TEAD4 and AP-1, resulting in sustained or enhanced oncogenic signaling. This adaptive response may limit the therapeutic efficacy of selective YAP1 inhibition. In contrast, dual inhibition of YAP1 and TAZ suppresses downstream transcriptional programs and promotes tumor suppression, highlighting the potential advantage of combined targeting strategies in GCPMs.

## Data Availability

Not applicable.
